# When educators are locked down: transitioning an international faculty development program from in-person to online during the COVID-19 pandemic in China

**DOI:** 10.12688/mep.19322.1

**Published:** 2022-09-27

**Authors:** Jonathan Lio, H. Barrett Fromme, Hongmei Dong, Ivy Jiang, Renslow Sherer

**Affiliations:** 1 Medicine, University of Chicago, Chicago, IL, 60637, USA; 2 Pediatrics, University of Chicago, Chicago, Illinois, 60637, USA

**Keywords:** COVID-19, medical education, international medical education, faculty development, curriculum

## Abstract

**Introduction:** The coronavirus disease 2019 (COVID-19) pandemic forced international faculty development programs in medical education to forgo in-person activities and transition to online learning. We sought to examine changes in Chinese medical educators’ evaluations of our faculty development program as it transitioned from in-person intensive to online longitudinal due to the pandemic.

**Methods:** A 30-item program evaluation and self-assessment of curriculum development and teaching skills was sent to our 2020 cohort. Results were compared to our 2019 cohort. We analyzed data using t-tests to compare means and chi-square test for categorical variables.

**Results:** We found that trainees in both cohorts rated the program highly with regard to overall program quality and self-assessed learning outcomes. Comparison of cohorts also showed similar growth in pre- and post-training assessment. However, the 2020 cohort rated their relationships with instructors and peers less strongly than the 2019 cohort.

**Conclusions:** Despite the rapid transition to online learning due to social distancing measures, trainees were as confident in the skills they learned as the prior in-person participants. Time zone differences placed additional restrictions on the implementation of the training program, which affected the amount of face-to-face interaction time available.

## Introduction

The coronavirus disease 2019 (COVID-19) pandemic dramatically altered business as usual for medical education ^1^. With social distancing as the most effective way to prevent transmission of infections in lieu of a vaccine, in-person activities were abandoned in favor of remote learning when possible, and medical schools have had to adapt students’ learning experiences based on the availability of PPE and local transmission rates. Likewise, faculty development programs have had to forgo in-person activities and transition to online learning ^2– 4^.

There is a paucity of literature regarding the influence of the pandemic on faculty development in medical education, but some have focused on aspects of online education, including decreased resistance from faculty to online programs ^5^, the degree of participation from trainees with online workshops ^6^, and the ease of bringing faculty from widespread regions together ^7^. None have discussed the impact of the online transition of international faculty development programs on training outcomes.

The University of Chicago, United States, has provided assistance for faculty development for Wuhan University, China, in the reformation of its medical curricula since 2008 ^8^. In 2018, the International Medical Educators Program (IMEP) was created to offer Wuhan faculty an immersive two-week training program in medical education on the University of Chicago campus. Due to the emergence of severe acute respiratory syndrome coronavirus 2 (SARS-CoV-2) in Wuhan and ensuing pandemic in 2020, this on-site training program was suspended, and the medical education program was quickly transitioned to an online program over Zoom.

The purpose of this study was to examine changes in Chinese medical educators’ evaluations of our faculty development program as it transitioned from in-person intensive to online longitudinal due to the COVID-19 pandemic, particularly with regard to program quality and self-assessed training outcomes.

## Methods

### Ethics

The Wuhan University Health Science Center first approved this study on 12 ^th^ August 2009 (IRB09126B). The University of Chicago Institutional Review Board approved an amendment to the study on 1 ^st^ September 2022 (IRB09126B). Signed consent and oral consent were waived.

### Training program description

IMEP was originally designed as a two-week intensive program at University of Chicago focused on curriculum development and teaching skills for Chinese medical educators. The program used a mixture of methods including lectures, observation, and hands-on experiences, and provided mentorship for individual curricular projects. Applicants were screened for English proficiency before matriculation. In the fall of 2020, IMEP transitioned to a 15-week program conducted over Zoom. While the content of training remained largely the same between 2019 and 2020 with the exception of hands-on topics such as simulation and teaching rounds observations, the total interactive face-to-face time decreased from 60 hours to 28 hours. 2020 trainees were aware of these changes before applying to the program. Despite the lengthened program duration, the 13-hour time difference limited possible interaction time to one hour on a given day, and due to the continued responsibilities of the physician educators during the pandemic, we scheduled only 1-2 sessions each week. With the reduction in total time available for interaction, we converted the curriculum development portion of the program to videos and homework assignments that participants could complete on their own time.

### Survey instrument

Each participant of the 2019 and 2020 cohorts of IMEP was sent a link by text, to an anonymous, electronic 43-item survey on DocumentStar, a Chinese survey platform, after the completion of the program. The survey was not tested before the study. The survey was conducted on 19 ^th^ July 2019 for the 2019 cohort and from 20 ^th^ December 2020 to 22 ^nd^ February 2021 for the 2020 cohort. Reminders were sent by program administrator Ivy Jiang via group text and email, until the response rate on DocumentStar reached 100%. Participants’ email and ID for texting were obtained when they registered for the training program. The survey asked participants to rate 36 statements pertaining to program evaluation and self-assessment of curriculum development and teaching skills (5-point Likert scale from “strongly disagree” to “strongly agree”). Self-assessment items included both retrospective pre- and post-training assessment. The remaining items asked for demographic information and open-ended responses. We defined academic seniority as associate professor or professor. The survey can be found as *Extended data* (Lio *et al*., 2022).

### Data analysis

We used paired sample t-test and independent sample t-tests to compare means and chi-squared test to compare categorical variables. The data were analyzed using Stata MP 16 for Windows (Stata Corp, College Station, Texas).

## Results

The survey response rate for IMEP both in 2019 and 2020 was 100% (29/29) (Lio *et al*., 2022). Demographic characteristics are in Table 1. We nearly doubled our class size in 2020 due to the ease of accommodating more trainees with the virtual format. There were no significant differences between participants of the two classes in terms of gender (60% male vs 79% female, p=0.278) and academic seniority (70% professor or associate professor vs 63% lecturer or other, p=.713). The representation of specialties shifted from more obstetrics and gynecology in 2019 to heavier weighting of internal medicine in 2020.

**Table 1.** Demographic characteristics of International Medical Educators Program (IMEP) participants.

Variable	2019 Median or N (%)	2020 Median or N (%)
Participants	10 (100%)	19 (100%)
Age	47.5	40
Female gender	6 (60.0)	15 (78.9)
Academic rank		
Senior	6 (60.0)	12 (63.2)
Junior	3 (30.0)	7 (36.8)
Location		
Wuhan	6 (60.0)	9 (47.4)
Beijing	3 (30.0)	10 (52.6)
Other	1 (10.0)	0 (0)
Specialty		
Internal Medicine	1 (10.0)	8 (42.1)
Ob & Gyn	3 (30.0)	4 (21.1)
Pediatrics	2 (20.0)	1 (5.3)
Surgery	2 (20.0)	2 (10.5)
Other	2 (20.0)	4 (21.1)

A comparison of program evaluation responses from participants in 2019 and 2020 is shown in Table 2. Trainees of both cohorts rated the program highly with respect to program quality and appropriateness of content and teaching methods. Although scores were high for every item, the 2019 cohort was more confident in being a better medical educator because of the program (statement 2). They also formed more meaningful relationships with instructors and planned to collaborate more with peers than the 2020 cohort (statements 6 and 8).

**Table 2.** General program feedback survey results for 2019 and 2020.

Statement	2019	2020
1. The training program was of high quality.	5	4.84
2. I will be a better medical educator because of this program.	4.9	4.53 *
3. The training program content was appropriate to my needs.	4.7	4.63
4. The instructional methods were appropriate for their respective content.	4.7	4.63
5. I could understand the training content well even though English is not my first language.	4.2	4.21
6. I have formed meaningful relationships with instructors of the training program.	4.6	3.95 *
7. The relationships I have formed with peer trainees during this training are valuable to me.	4.8	4.53
8. I plan to collaborate with my peer trainees in the future.	4.8	4.21 *
9. I work with other medical educators (e.g. course directors) with whom I will share the contents of this training.	4.7	4.58
10. The skills I have learned in the training program are easily adaptable to my work setting in China.	4.5	4.26

*p<.05

Trainees in both cohorts were confident in using skills they learned from the program and in implementing their individual curricular projects ( Table 3). There was a significant difference between the cohorts for five of the curriculum development and teaching skills (statements 14, 16, 20, 22, 23), but a comparison of pre-training and post-training improvement did not show a significant difference for any of these items between the two years. Self-assessment of pre-training and post-training skills for each year demonstrated a significant improvement for every item, with a mean increase in Likert scale rating of 1.68 (p<0.001) for the class of 2019 and 1.45 (p<0.001) for the class of 2020. All 2020 trainees except one preferred in-person training to online training. Seven individuals wrote in the free response section that classes were too late at night.

**Table 3.** Curriculum development and teaching skills self-assessment for 2019 and 2020.

Statement	2019 post	2020 post	2019 Pre-post Change	2020 Pre-post Change
11. I can identify a health problem that requires a new curriculum.	4.7	4.37	1.2	1.32
12. I can assess potential learners of a new curriculum.	4.7	4.32	1.6	1.53
13. I can write educational objectives for a new curriculum.	4.7	4.68	2	1.89
14. I can select appropriate educational strategies for a new curriculum.	4.7	4.16 *	2.2	1.58
15. I can gather support and necessary resources to develop and implement a new curriculum.	4.7	4.42	1.6	1.21
16. I can develop an evaluation plan for a new curriculum.	4.8	4.32 *	1.9	1.63
17. I can disseminate my curriculum as scholarship.	4.4	3.95	1.8	1.21
18. I am confident I can implement the education project I have chosen for this training program.	4.4	4.32	1.6	1.42
19. I understand basic learning theory.	4.3	4.21	2	2.05
20. I can deliver lectures that help my learners learn effectively.	4.9	4.42 *	1.1	1.11
21. I am comfortable using the skills of the One Minute Preceptor.	4.7	4.37	2.7	1.37
22. I can give effective feedback to my learners.	4.7	4.26 *	1.3	1.53
23. I am an effective clinical teacher.	4.9	4.32 *	.8	.89

*p<.05

## Discussion

Despite the disruption from the COVID-19 pandemic and rapid transition of our Chinese faculty development program from in-person to online, participants rated the overall program quality highly, similar to the previous year’s participants. Trainees found the educational content and methods used in the program appropriate to their needs. They were also confident in using curriculum development and teaching skills as shown by the high scores across all 13 self-assessment items. These results are consistent with literature showing minimal or negligible differences between online learning and traditional approaches ^9^.

However, trainees’ perception of the strength of their education community was weaker in the online group compared to the in-person group. In particular, they rated both the strength of their relationships with instructors and plans to collaborate with other trainees lower in 2020 (statements 6 and 8). Intentional community building is increasingly seen as an integral component of successful longitudinal faculty development ^10^, but building virtual communities of practice is rarely discussed in health professions and deserves more focus ^11, 12^.

Logistical considerations may also have affected the cohesion of the group. Although the socially distanced virtual setting allowed us to accommodate more trainees with ease, it posed other challenges. Some of the most successful international models use a short in-person period to foster community growth before moving online for the rest of the year ^13^, yet the pandemic did not allow for this. Nearly all of our 2020 trainees preferred training to be in-person, whether in China or Chicago. In addition, over one third of trainees thought classes were too late, even though there was only one feasible time slot for face-to-face interaction given the time zone difference. Community was even more difficult to achieve with overall face-to-face time halved from the previous year. For subsequent training years that are virtual as well, we plan to allocate more face-to-face time from lecture to discussion, and assign group projects to encourage more interaction and mutual learning ^11, 14^.

Another consequence of the pandemic was that we increased the duration of the program to 15 weeks, which allowed participants more time to process training content, as well as receive longitudinal mentorship for their individual education projects. However, this did not translate to increased perception of learning for any of the survey items compared to 2019, and participants were just as confident in implementing their education projects as the previous year. Notwithstanding the self-assessment data, we will be following up with trainees to evaluate the quality of the projects after they are implemented and compare differences between the cohorts ^15^.

A major limitation of this study is that it only includes data from a single faculty development program, and validity of the data is limited by small sample sizes. Additionally, the variety of changes to the program due to disruption from the pandemic limits the interpretation of results in other contexts.

The COVID-19 pandemic forced this international faculty development program to transition urgently from in-person to online. Overall, trainees were satisfied with the program and rated learning as high as the previous year, although relationships with instructors and peers were weaker. Educators at other institutions working with international faculty development programs during the pandemic need to consider the optimization of limited interactive time due to time zone differences, and pay attention to community dynamics. For future virtual iterations of IMEP, we will continue using asynchronous materials to supplement our face-to-face virtual sessions, but we plan to increase time for discussion and assign group projects to help build community among our trainees and instructors.

## Data Availability

Mendeley Data: IMEP Survey 2019/20. https://doi.org/10.17632/kdjty6js4f.2 (Lio *et al*., 2022). This project contains the following underlying data: IMEP Survey 2019-20.xlsx Mendeley Data: IMEP Survey 2019/20. https://doi.org/10.17632/kdjty6js4f.2 (Lio *et al*., 2022). This project contains the following extended data: Post-Training Survey Blank.docx Data are available under the terms of the Creative Commons Attribution 4.0 International license (CC-BY 4.0).
