# When educators are locked down: transitioning an international faculty development program from in-person to online during the COVID-19 pandemic in China

**DOI:** 10.12688/mep.19322.2

**Published:** 2023-08-10

**Authors:** Jonathan Lio, H. Barrett Fromme, Hongmei Dong, Ivy Jiang, Renslow Sherer

**Affiliations:** 1 Medicine, University of Chicago, Chicago, IL, 60637, USA; 2 Pediatrics, University of Chicago, Chicago, Illinois, 60637, USA

**Keywords:** COVID-19, medical education, international medical education, faculty development, curriculum

## Abstract

**Introduction:** The coronavirus disease 2019 (COVID-19) pandemic forced international faculty development programs in medical education to forgo in-person activities and transition to online learning. We sought to examine changes in international medical educators’ evaluations of our faculty development program as it transitioned due to the pandemic.

**Methods:** We compared survey responses from participants in our International Medical Educators Program between 2019 (in-person) and 2020 (online). The 43-item survey addressed aspects such as program evaluation and self-assessment of curriculum development and teaching skills. We analyzed data using t-tests to compare means and chi-square test for categorical variables, and performed thematic analysis of open-ended responses.

**Results:** We found that trainees in both cohorts rated the program highly with regard to overall program quality and self-assessed learning outcomes, but the 2019 group reported stronger relationships with peers and instructors. Some scores for self-assessed outcomes were lower for the 2020 class, but no statistically significant differences were found in pre- and post- training scores between the two cohorts. Four themes emerged from the feedback: positive program utility, IMEP as an example of good curriculum design, timing issues, and online learning environment challenges.

**Conclusions:** Despite pandemic challenges, the transition to online faculty development was favorably evaluated, with high confidence in the applicability of learned skills. Future efforts should focus on fostering community and optimizing interaction times to enhance learning experiences. The study contributes insights for global medical education communities in pandemic circumstances.

## Introduction

The coronavirus disease 2019 (COVID-19) pandemic dramatically altered the landscape of medical education worldwide in 2020 ^1, 2^. With social distancing as the most effective way to prevent transmission of infections in lieu of a vaccine, in-person activities were abandoned in favor of remote learning when possible, and medical schools have had to adapt students’ learning experiences based on the availability of PPE and local transmission rates. Likewise, faculty development programs have had to forgo in-person activities and transition to online learning ^3– 5^.

This abrupt transition to online learning brought a set of considerable challenges. Educators grappled with delivering clinical skills, teaching remotely, and handling large groups of students in online settings ^6^. These issues were exacerbated by uneven institutional support, influenced by educators’ individual technological literacy, inadequate resource planning, and usability problems like screen resolution and audio quality ^7^. Despite these hurdles, perceptions of online teaching demonstrated a broad spectrum: while less than half of educators and learners from one study thought online training could match the quality of traditional face-to-face courses, a significant portion saw its potential beyond the pandemic ^8^. Notably, this shift also highlighted emerging pedagogical opportunities, as educators called for increased support in formats such as online lectures, collaborative work, live broadcasts, and chats ^9^.

Literature on the pandemic’s influence on faculty development in medical education is limited. Some studies have addressed facets of online education like reduced resistance from faculty to online programs ^10^, the degree of participation from trainees with online workshops ^11^, and the ease of bringing faculty from widespread regions together ^12^. However, the impact of the online transition of international faculty development programs on training outcomes has not been discussed.

### Training program context

The University of Chicago, United States, has provided assistance for faculty development for Wuhan University, China, in the reformation of its medical curricula since 2008 ^13^. This collaborative effort culminated in the creation of the International Medical Educators Program (IMEP) in 2018. IMEP was designed to provide Wuhan faculty members with an immersive two-week training program in medical education at the University of Chicago campus. However, due to the emergence of severe acute respiratory syndrome coronavirus 2 (SARS-CoV-2) in Wuhan and ensuing pandemic in 2020, this on-site training program was suspended, and the program was quickly transitioned to an online format delivered through Zoom.

The transition to a virtual platform in 2020 necessitated a few adaptations to our training program. We managed to keep the core of the program consistent with the 2019 version, except for the unavoidable exclusion of hands-on topics such as simulations and teaching rounds observations. However, the consistency of the program was upheld as the instructors and their respective course content remained the same across cohorts. To accommodate the 13-hour time difference and ongoing responsibilities of physician educators during the pandemic, we restructured our schedule to only hold 1–2 one-hour sessions a week. Consequently, the total interactive face-to-face time was reduced from 60 hours to 28 hours.

### Study purpose

The purpose of this study was to examine changes in international medical educators’ perceptions of one faculty development program as it transitioned from in-person to online due to the COVID-19 pandemic. Specifically, we aimed to analyze program quality and self-assessed training outcomes to provide insights applicable to global medical education communities.

## Methods

### Ethics

The Wuhan University Health Science Center first approved this study on 12 ^th^ August 2009 (IRB09126B). The University of Chicago Institutional Review Board approved an amendment to the study on 1 ^st^ September 2022 (IRB09126B). Signed consent and oral consent were waived.

### Training program description

IMEP began as a two-week intensive program at University of Chicago, with an aim to improve curriculum development and teaching skills for international medical educators. However, the necessity for social distancing and the challenge of a 13-hour time difference led to the transition into a 15-week program. Given the reduced interactive time, we adapted the curriculum development section of the program into video modules and homework assignments, which allowed participants to progress at their own pace outside the confines of group sessions. To facilitate discussion and feedback on participants’ individual curriculum development projects, the group convened on Zoom every two weeks.

Initially, IMEP used a broad range of teaching methods including lectures, observation, and interactive experiences. The advent of the pandemic led to the removal of interactive simulation and observation experiences from the program. However, the extension of the program to 15 weeks enabled longitudinal mentorship for individual curriculum projects. Our lecture topics encompassed a wide array of subjects, including learning theory, competency-based medical education, the one-minute preceptor approach, providing feedback, PowerPoint design, lecture skills, role modeling, and reflection. These lectures were delivered via Zoom for a duration of one hour, followed by a 15-minute breakout session for small group discussions.

To ensure smooth communication and understanding, applicants underwent an English proficiency screening before matriculation.

### Survey instrument

Upon completing the program, all trainees from the 2019 and 2020 IMEP cohorts were provided a link via text message to a 43-item anonymous survey hosted on DocumentStar, a Chinese survey platform. The survey was not tested before the study. The survey was conducted on 19 ^th^ July 2019 for the 2019 cohort and from 20 ^th^ December 2020 to 22 ^nd^ February 2021 for the 2020 cohort. Reminders were sent by the program administrator via group text and email, until the response rate on DocumentStar reached 100%. Participants’ email and ID for texting were obtained when they registered for the training program. The survey asked participants to rate 36 statements pertaining to program evaluation and self-assessment of curriculum development and teaching skills (5-point Likert scale from “strongly disagree” to “strongly agree”). Self-assessment items included both retrospective pre- and post-training assessment. The remaining items asked for demographic information and open-ended responses. We defined academic seniority as associate professor or professor. The survey can be found as *Extended data* (Lio *et al*., 2022).

### Data analysis

We used paired sample t-test and independent sample t-tests to compare means and chi-squared test to compare categorical variables. The data were analyzed using Stata MP 16 for Windows (Stata Corp, College Station, Texas). We also conducted a thematic analysis of the responses to open-ended questions, which involved an iterative process of reading and coding the responses to identify themes.

## Results

The survey response rate for IMEP both in 2019 and 2020 was 100% (10/10 and 19/19 respectively) (Lio *et al*., 2022). Demographic characteristics are in Table 1. We nearly doubled our class size in 2020 due to the ease of accommodating more trainees with the virtual format. There were no significant differences between participants of the two classes in terms of gender (60% female in 2019 vs 79% female in 2020, p=0.278) and academic seniority (70% professor or associate professor in 2019 vs 63% in 2020, p=.713). The representation of specialties shifted from more obstetrics and gynecology in 2019 to heavier weighting of internal medicine in 2020.

**Table 1.** Demographic characteristics of International Medical Educators Program (IMEP) participants.

Variable	2019 Median or N (%)	2020 Median or N (%)
Participants	10 (100%)	19 (100%)
Age	47.5	40
Female gender	6 (60.0)	15 (78.9)
Academic rank		
Senior	6 (60.0)	12 (63.2)
Junior	3 (30.0)	7 (36.8)
Location		
Wuhan	6 (60.0)	9 (47.4)
Beijing	3 (30.0)	10 (52.6)
Other	1 (10.0)	0 (0)
Specialty		
Internal Medicine	1 (10.0)	8 (42.1)
Ob & Gyn	3 (30.0)	4 (21.1)
Pediatrics	2 (20.0)	1 (5.3)
Surgery	2 (20.0)	2 (10.5)
Other	2 (20.0)	4 (21.1)

A comparison of program evaluation responses from participants in 2019 and 2020 is shown in Table 2. Trainees of both cohorts rated the program highly with respect to program quality and appropriateness of content and teaching methods. Although scores were high for every item, the 2019 cohort was more confident in being a better medical educator because of the program (statement 2). They also formed more meaningful relationships with instructors and planned to collaborate more with peers than the 2020 cohort (statements 6 and 8).

**Table 2.** General program feedback survey results for 2019 and 2020.

Statement	2019	2020
1. The training program was of high quality.	5	4.84
2. I will be a better medical educator because of this program.	4.9	4.53 *
3. The training program content was appropriate to my needs.	4.7	4.63
4. The instructional methods were appropriate for their respective content.	4.7	4.63
5. I could understand the training content well even though English is not my first language.	4.2	4.21
6. I have formed meaningful relationships with instructors of the training program.	4.6	3.95 *
7. The relationships I have formed with peer trainees during this training are valuable to me.	4.8	4.53
8. I plan to collaborate with my peer trainees in the future.	4.8	4.21 *
9. I work with other medical educators (e.g. course directors) with whom I will share the contents of this training.	4.7	4.58
10. The skills I have learned in the training program are easily adaptable to my work setting in China.	4.5	4.26

*p<.05

Trainees in both cohorts were confident in using skills they learned from the program and in implementing their individual curricular projects ( Table 3). There was a significant difference between the cohorts for five of the curriculum development and teaching skills (statements 14, 16, 20, 22, 23), but a comparison of pre-training and post-training improvement did not show a significant difference for any of these items between the two years. Self-assessment of pre-training and post-training skills for each year demonstrated a significant improvement for every item, with a mean increase in Likert scale rating of 1.68 (p<0.001) for the class of 2019 and 1.45 (p<0.001) for the class of 2020. All 2020 trainees except one preferred in-person training to online training.

**Table 3.** Curriculum development and teaching skills self-assessment for 2019 and 2020.

Statement	2019 post	2020 post	2019 Pre-post Change	2020 Pre-post Change
11. I can identify a health problem that requires a new curriculum.	4.7	4.37	1.2	1.32
12. I can assess potential learners of a new curriculum.	4.7	4.32	1.6	1.53
13. I can write educational objectives for a new curriculum.	4.7	4.68	2	1.89
14. I can select appropriate educational strategies for a new curriculum.	4.7	4.16 *	2.2	1.58
15. I can gather support and necessary resources to develop and implement a new curriculum.	4.7	4.42	1.6	1.21
16. I can develop an evaluation plan for a new curriculum.	4.8	4.32 *	1.9	1.63
17. I can disseminate my curriculum as scholarship.	4.4	3.95	1.8	1.21
18. I am confident I can implement the education project I have chosen for this training program.	4.4	4.32	1.6	1.42
19. I understand basic learning theory.	4.3	4.21	2	2.05
20. I can deliver lectures that help my learners learn effectively.	4.9	4.42 *	1.1	1.11
21. I am comfortable using the skills of the One Minute Preceptor.	4.7	4.37	2.7	1.37 *
22. I can give effective feedback to my learners.	4.7	4.26 *	1.3	1.53
23. I am an effective clinical teacher.	4.9	4.32 *	.8	.89

*p<.05

### Participant feedback and thematic analysis

Participants' open-ended written feedback on the program was analyzed, and four main themes emerged: the positive learning experience and utility of the program, timing concerns, the online learning environment, and interaction and discussion needs. The latter two themes were noted only by the 2020 cohort.

Utility of the program: Participants valued the program and its utility in practical application, including curriculum design and the application of educational theory and specific teaching strategies and skills, such as assessment methods and feedback skills. One trainee noted, "It is very useful to me to design curriculum... And these information is valuable to apply in the daily ward trainings, lectures, and evaluation for teachers (sic)."

IMEP as an example of good curriculum design: A theme emerged emphasizing IMEP as an example of good curriculum design. The trainees commended the balance of educational theory and practical techniques, and they valued the application of these concepts to their own professional contexts. Participants described IMEP as a "standard model of future curriculum development" and as a "beacon of effective curriculum design". One trainee encapsulated these views, saying, "The training program itself shows us how to design courses, implement courses, choose teaching strategies, evaluate, etc. which is very helpful for us to understand the design of medical courses."

Timing concerns: Many comments pointed to issues related to timing of the classes, suggesting it affected their ability to fully engage with the material and discussions. Seven participants expressed issues with late-night classes due to time-zone differences, especially after the end of daylight savings time. For example, one trainee wrote, "too late at night because of the time difference."

Online learning environment: Participants had mixed feelings about the online learning environment. While some appreciated the necessity of it due to pandemic circumstances, others highlighted challenges, including issues with engagement and focus. A few participants expressed a desire for more interactive and practical components, and one commented on the inefficiency of online group discussions. One participant noted, "being online is a little bit hard to focus, especially when we are still being bothered by a lot of things in the clinic." Another wrote, “All the professors gave us wonderful talk although on-line course is lack of interaction between instructors and learners (sic).”

## Discussion

The COVID-19 pandemic led to the unforeseen transition of our international faculty development program from an in-person format to an online environment. This study explores the less examined area of the pandemic’s influence on international faculty development in medical education, and offers critical insights for the wider medical education community.

In spite of the shift to an online platform, participants rated the overall program quality favorably, echoing the high evaluations from the previous year’s cohort. Trainees indicated that the program’s educational content and instructional methods met their needs. Their confidence in using curriculum development and teaching skills was further demonstrated by high scores across all 13 self-assessment items. Although some scores in the 2020 class were slightly lower than those of the 2019 class, the comparison of pre- and post-program scores revealed no statistically significant differences. These findings are consistent with existing literature suggesting minimal or negligible differences between online learning and traditional approaches ^14^. However, it is worth noting the lower scores may indicate a trend that needs monitoring.

The analysis of trainees’ feedback also revealed certain challenges within this new format. Trainees’ perception of the strength of their education community was weaker in the online group compared to the in-person group. This was particularly evident in the lower ratings given to the quality of relationships with instructors and prospects for future collaboration with other trainees (statements 6 and 8) in 2020. Some individuals also expressed in their open-ended responses a desire for a higher degree of interactivity and engagement. While the 2020 cohort of medical educators maintained confidence that they would be better educators due to the training program, they were slightly less confident compared to their 2019 counterparts. These insights highlight the potential challenges of fostering an online learning environment that encourages peer-to-peer and mentor-mentee interactions. Intentional community building is increasingly seen as an integral component of successful longitudinal faculty development ^15^, and these findings underscore the importance of further optimizing online teaching methods to facilitate interactive engagement, foster meaningful relationships, and stimulate future collaborations among participants and instructors. Despite its importance, building virtual communities of practice is seldom discussed in health professions and deserves more focus ^16, 17^.

Logistical considerations may also have affected the cohesion of the group. Although the socially distanced virtual setting allowed us to accommodate more trainees with ease, it posed other challenges. Some of the most successful international models use a short in-person period to foster community growth before moving online for the rest of the year ^18^, yet the pandemic did not allow for this. Nearly all of our 2020 trainees preferred training to be in-person, whether in China or Chicago. In addition, over one third of trainees thought classes were too late, even though there was only one feasible time slot for face-to-face interaction given the time zone difference. Community was even more difficult to achieve with overall face-to-face time halved from the previous year. For subsequent training years that are virtual as well, we plan to allocate more face-to-face time from lecture to discussion, and assign group projects to encourage more interaction and mutual learning ^16, 19^.

Another consequence of the pandemic was that we increased the duration of the program to 15 weeks, which allowed participants more time to process training content, as well as receive longitudinal mentorship for their individual education projects. However, this did not translate to increased perception of learning for any of the survey items compared to 2019, and participants were just as confident in implementing their education projects as the previous year. Notwithstanding the self-assessment data, we will be following up with trainees to evaluate the quality of the projects after they are implemented and compare differences between the cohorts ^20^.

### Best practices

Based on our experiences with the International Medical Educators Program (IMEP), we outline a set of best practices for transitioning an international faculty development program to an online format:

1. Consider Time Zone Differences: Adjust the schedule to consider the time zone differences for international participants to maximize active engagement.
2. Increase Interaction: Implement more interactive and engaging activities to foster active learning, even in a virtual setting. This might include group discussions, breakout rooms for small group work, and interactive quizzes or polls during sessions.
3. Foster Community: Implement strategies to foster a sense of community among participants, despite not being physically present. This might involve setting up virtual social events, allowing for casual conversation before and after sessions, and using collaboration tools for group projects.
4. Asynchronous Learning: Make use of asynchronous learning tools and resources, such as recorded lectures, discussion boards, and learning management systems. This can supplement synchronous sessions and accommodate participants in various time zones.
5. Flexibility: Be flexible and ready to adapt the program as needed, based on feedback from participants, changes in public health conditions, and any technical challenges that might arise.
6. Evaluate and Iterate: Regularly collect and analyze feedback from participants to understand their learning experience, identify any issues or challenges, and make necessary improvements to the program.

By implementing these practices, we believe that international faculty development programs can successfully transition to an online format, maintaining high levels of learning, engagement, and community, even amidst challenging circumstances of social distancing such as the COVID-19 pandemic.

### Limitations

A major limitation of this study is that it only includes data from a single faculty development program, and validity of the data is limited by small sample sizes. Additionally, the variety of changes to the program due to disruption from the pandemic limits the interpretation of results in other contexts. Another limitation was that the 2020 course was not modified to address new skills required of medical educators, such as the use of online teaching tools and online communication skills.

## Conclusion

The COVID-19 pandemic forced this international faculty development program to transition urgently from in-person to online. Overall, trainees were satisfied with the program and rated learning as high as the previous year, although they reported weaker relationships with instructors and peers. When planning international faculty development programs during a pandemic, educators need to consider optimizing limited interactive time due to time zone differences, while also fostering a sense of community. For future virtual iterations of IMEP, we will continue using asynchronous materials to supplement our face-to-face virtual sessions, but we plan to increase time for discussion and assign group projects to help build community among our trainees and instructors.

## Data Availability

Mendeley Data: IMEP Survey 2019/20. https://doi.org/10.17632/kdjty6js4f.2 (Lio *et al*., 2022). This project contains the following underlying data: IMEP Survey 2019-20.xlsx Mendeley Data: IMEP Survey 2019/20. https://doi.org/10.17632/kdjty6js4f.2 (Lio *et al*., 2022). This project contains the following extended data: Post-Training Survey Blank.docx Data are available under the terms of the Creative Commons Attribution 4.0 International license (CC-BY 4.0).
