# Supplementary material for: When educators are locked down: transitioning an international faculty development program from in-person to online during the COVID-19 pandemic in China
Source: MedEdPublish (2016). 2023 Aug 10;12:59. Originally published 2022 Sep 27. [Version 2] doi: 10.12688/mep.19322.2 (PMC10587660; doi:10.12688/mep.19322.2)
Supplement: Peer review history

# Reviewer report: Reviewer response for version 2

**DOI:** 10.21956/mep.21161.r34485

**Published:** 2023-09-28

**Authors:** Samar A Ahmed

**Affiliations:** 1 Dubai College, Dubai, Dubai, United Arab Emirates; 2 Ain Shams University, Cairo, Egypt

The authors have corrected the missing parts now.

Have any limitations of the research been acknowledged?

No

Is the work clearly and accurately presented and does it cite the current literature?

Partly

If applicable, is the statistical analysis and its interpretation appropriate?

Yes

Are all the source data underlying the results available to ensure full reproducibility?

No

Is the study design appropriate and is the work technically sound?

Partly

Are the conclusions drawn adequately supported by the results?

Partly

Are sufficient details of methods and analysis provided to allow replication by others?

No

Reviewer Expertise:

Medical education, program evaluation, contextualization of programs

I confirm that I have read this submission and believe that I have an appropriate level of expertise to confirm that it is of an acceptable scientific standard.

# Reviewer report: Reviewer response for version 2

**DOI:** 10.21956/mep.21161.r34486

**Published:** 2023-08-22

**Authors:** Rusnani Ab Latif

**Affiliations:** 1 Universiti Teknology MARA, Pulau Pinang, Malaysia

The authors have made the amendments based on the comments.

Have any limitations of the research been acknowledged?

Yes

Is the work clearly and accurately presented and does it cite the current literature?

Partly

If applicable, is the statistical analysis and its interpretation appropriate?

Yes

Are all the source data underlying the results available to ensure full reproducibility?

Yes

Is the study design appropriate and is the work technically sound?

Yes

Are the conclusions drawn adequately supported by the results?

Yes

Are sufficient details of methods and analysis provided to allow replication by others?

Yes

Reviewer Expertise:

1. Obstetric and gynaecilogy 2. Community 3. Women health 4. Midwifery 5. Nursing Education

I confirm that I have read this submission and believe that I have an appropriate level of expertise to confirm that it is of an acceptable scientific standard.

# Reviewer report: Reviewer response for version 1

**DOI:** 10.21956/mep.20705.r33731

**Published:** 2023-07-05

**Authors:** Samar A Ahmed

**Affiliations:** 1 Dubai College, Dubai, Dubai, United Arab Emirates; 2 Ain Shams University, Cairo, Egypt

The work, while done very meticulously, offers very limited generalizability of the results. The study question needs to be revised for this international portal and to suit its current readership. 

 The section under methods that describes the training program looks more like a context description that I think would better suit the introduction piece and leave this section for an actual description of how the program is currently conducted.

 The work is an evaluation study of a faculty development program in its new form that has been shifted to online and has extended its duration for the sake of compliance. I was looking for a governing criteria that individualized this program and that can be taken as a take home message for readers who are embarking on designing their own faculty development program but unfortunately I could not find this. 

 In order to make this piece better utilizable by readers I wonder if a section on limitations and best practices can be added for example.

Have any limitations of the research been acknowledged?

No

Is the work clearly and accurately presented and does it cite the current literature?

Partly

If applicable, is the statistical analysis and its interpretation appropriate?

Yes

Are all the source data underlying the results available to ensure full reproducibility?

No

Is the study design appropriate and is the work technically sound?

Partly

Are the conclusions drawn adequately supported by the results?

Partly

Are sufficient details of methods and analysis provided to allow replication by others?

No

Reviewer Expertise:

Medical education, program evaluation, contextualization of programs

I confirm that I have read this submission and believe that I have an appropriate level of expertise to state that I do not consider it to be of an acceptable scientific standard, for reasons outlined above.

# Reviewer report: Reviewer response for version 1

**DOI:** 10.21956/mep.20705.r33728

**Published:** 2023-06-20

**Authors:** Rusnani Ab Latif

**Affiliations:** 1 Universiti Teknology MARA, Pulau Pinang, Malaysia

- The inclusion and exclusion criteria for the study need to be discussed here.
- Write the implications of the study.
- Write the strengths of the study.
- How do the findings add to the body of scientific knowledge on the issue?
- References 8, 9, 10, 13, 14,and 15 are more than 10 years old, it is better to replace them with updated references.

Have any limitations of the research been acknowledged?

Yes

Is the work clearly and accurately presented and does it cite the current literature?

Partly

If applicable, is the statistical analysis and its interpretation appropriate?

Yes

Are all the source data underlying the results available to ensure full reproducibility?

Yes

Is the study design appropriate and is the work technically sound?

Yes

Are the conclusions drawn adequately supported by the results?

Yes

Are sufficient details of methods and analysis provided to allow replication by others?

Yes

Reviewer Expertise:

1. Obstetric and gynaecilogy 2. Community 3. Women health 4. Midwifery 5. Nursing Education

I confirm that I have read this submission and believe that I have an appropriate level of expertise to confirm that it is of an acceptable scientific standard, however I have significant reservations, as outlined above.

# Reviewer report: Reviewer response for version 1

**DOI:** 10.21956/mep.20705.r33236

**Published:** 2023-06-02

**Authors:** Adrienne Torda

**Affiliations:** 1 The University of New South Wales, New South Wales, Australia

Although the design of the study and the tool used (and the high response rate) would all be appropriate under normal circumstances, as a result of the COVID pandemic and the rapid switch to online teaching and pivot in how students’ learn even in the clinical environment, many new challenges were faced in medical education. Some of these are outlined in these recent publications.

  

- Chan *et al.* (2022 ^1^).
- Liu *et al.* (2022 ^2^).
- Motte-Signoret *et al.* (2021 ^3^).
- Bastos *et al. *(2022 ^4^).
- Herrmann-Werner *et al.* (2021 ^5^).

There are a number of limitations which aren’t mentioned.

 What isn’t stated is whether the same content was delivered in the 2020 online course, or whether it was modified to address the new skills required of medical educators. These include the following:

- Use of technology and online teaching platforms/tools

- Online communication skills

- Creation of online learning resources

- Synchronous online teaching and online use of learning activities

Also, the trend for all scores for both the General feedback and the Curriculum development skills statements, was that they were lower in 2020. In the general feedback, there were significantly lower scores for statements 2, 6 and 8. and For Curriculum development skills statements, 5 statements, there was a significant difference. It is quite telling that the lowest response was for the statement around formation of meaningful relationships (the next was plans for collaboration), which may reflect a failing of the transition to this online professional development program. There was also a significant lowering of the score for the self-confidence in delivering a lecture that helps students learn effectively. This is only discussed tangentially in relation to poorer community building.

 It is impossible to speculate on the causes for this without knowing whether the content has been changed at all or how well skilled the facilitators of the new online course were themselves in the skills listed above. In 2020, many of us were still using new techniques for the first time. This may well have affected the delivery of this course.

 This study would benefit from free text qualitative feedback if this was collected. They mention that the students found the timing difficult and that they preferred face to face, but only in the discussion, not as a thematic review of qualitative data (if this was done).

 Some pluses of this mode of delivery, such as enabling bigger cohorts. this is mentioned, but in this group it looks as if the same number (29) were enrolled. So, this needs to be clarified. This size may well require a different approach, such as the creation of smaller online breakout groups to encourage interaction and enable the creation of ‘community’ and more meaningful relationships with colleagues.

Have any limitations of the research been acknowledged?

Partly

Is the work clearly and accurately presented and does it cite the current literature?

Partly

If applicable, is the statistical analysis and its interpretation appropriate?

Partly

Are all the source data underlying the results available to ensure full reproducibility?

Partly

Is the study design appropriate and is the work technically sound?

Partly

Are the conclusions drawn adequately supported by the results?

No

Are sufficient details of methods and analysis provided to allow replication by others?

Yes

Reviewer Expertise:

medical education, professional development, online learning

I confirm that I have read this submission and believe that I have an appropriate level of expertise to state that I do not consider it to be of an acceptable scientific standard, for reasons outlined above.

## References

[1] : Medical teachers' experience of emergency remote teaching during the COVID-19 pandemic: a cross-institutional study. *BMC Med Educ* .2022;22(1) : 10.1186/s12909-022-03367-x 303 10.1186/s12909-022-03367-x 35449047PMC9021818

[2] : The Impact of Teacher Competence in Online Teaching on Perceived Online Learning Outcomes during the COVID-19 Outbreak: A Moderated-Mediation Model of Teacher Resilience and Age. *Int J Environ Res Public Health* .2022;19(10) : 10.3390/ijerph19106282 10.3390/ijerph19106282 35627819PMC9140542

[3] : Perception of medical education by learners and teachers during the COVID-19 pandemic: a cross-sectional survey of online teaching. *Med Educ Online* .2021;26(1) : 10.1080/10872981.2021.1919042 1919042 10.1080/10872981.2021.1919042 33871308PMC8079026

[4] : Solutions, enablers and barriers to online learning in clinical medical education during the first year of the COVID-19 pandemic: A rapid review. *Med Teach* .2022;44(2) : 10.1080/0142159X.2021.1973979 187-195 10.1080/0142159X.2021.1973979 34608845

[5] : Medical education in times of COVID-19: survey on teachers' perspectives from a German medical faculty. *GMS J Med Educ* .2021;38(5) : 10.3205/zma001489 Doc93 10.3205/zma001489 34286073PMC8256125
